# Relevance of Cognition and Emotion for Patient-Reported Quality of Life After Stroke in Working Age: An Observational Cohort Study

**DOI:** 10.3389/fneur.2022.869550

**Published:** 2022-04-25

**Authors:** Daniela Pinter, Simon Fandler-Höfler, Viktoria Fruhwirth, Lisa Berger, Gerhard Bachmaier, Susanna Horner, Sebastian Eppinger, Markus Kneihsl, Christian Enzinger, Thomas Gattringer

**Affiliations:** ^1^Department of Neurology, Research Unit for Neuronal Plasticity and Repair, Medical University of Graz, Graz, Austria; ^2^Department of Neurology, Medical University of Graz, Graz, Austria; ^3^Institute for Medical Informatics, Statistics and Documentation, Medical University of Graz, Graz, Austria; ^4^Division of Neuroradiology, Department of Radiology, Vascular and Interventional Radiology, Medical University of Graz, Graz, Austria

**Keywords:** quality of life, cognition, emotion, working-age, stroke

## Abstract

**Background:**

Patient-reported quality of life (QoL) may help to capture sequela of stroke more comprehensively. We aimed to investigate QoL in working age persons with ischemic stroke regarding impaired domains and identify factors associated with better QoL.

**Methods:**

We invited persons with stroke aged 18–55 years to participate in this prospective observational study. We assessed QoL and self-rated health using the EuroQol 5 Dimension questionnaire (EQ-5D) during hospital stay (baseline) and at 3-months follow-up (FU). Additionally, the National Institute of Health Stroke Scale (NIHSS), modified Rankin Scale (mRS), cognition (Montreal Cognitive assessment, MOCA), emotion (Hospital Anxiety and Depression Scale), and return to work were evaluated. We used hierarchical regression to identify predictors of QoL (self-rated health and QoL Index score) at FU.

**Results:**

We included 138 persons with stroke (mean age = 43.6 ± 10 years; 41% female; median admission NIHSS = 2), of whom 99 participated at FU. QoL Index and self-rated health were correlated with NIHSS, mRS, anxiety, and depression at both timepoints. Although 80% had favorable functional outcome at FU (mRS < 2), high proportions of these persons reported problems in the “Pain and/or Discomfort” (25.3%) and “Anxiety/Depression” (22.8%) dimensions. Only discharge NIHSS and baseline MOCA independently predicted self-rated health at FU. Female sex, higher discharge NIHSS, and higher baseline depression scores predicted worse QoL Index scores at FU.

**Conclusions:**

Three months post-stroke, working age persons with stroke frequently reported problems in dimensions not assessed by the routinely used mRS. Despite correlations between clinical scales and QoL, patient-reported outcomes and screening for cognition and emotion ensure a more comprehensive assessment of post-stroke consequences relevant for QoL.

## Introduction

Stroke at working age is on the rise, with ~10–15% of all ischemic strokes occurring in persons aged below 55 years (1, 2). These stroke survivors may live many years with detrimental effects in multiple health domains, such as physical, psychological, and/or social problems (3).

Some so-called “hidden” consequences of stroke (e.g., cognitive impairment, anxiety, depression) are not captured by routine clinical scales like the modified Rankin Scale (mRS) (4–6). Therefore, assessment of patient-reported outcome measures are increasingly recommended (7) to better capture diverse sequelae of stroke (5, 8).

To date, few studies have examined health-related quality of life (QoL) in working age persons with stroke (9, 10). Identification of factors influencing QoL in this patient group is essential to guide comprehensive clinical assessment and rehabilitation decisions and enable a more efficient and patient orientated (post-stroke) health care.

Prior studies highlighted physical impairment and psychological factors, such as depression, anxiety or fatigue to be associated with QoL in working age persons with stroke (11, 12). Recent work showed that in older persons with stroke, depression and cognitive impairment are independent predictors of participation and independence (13).

In this prospective, observational, and single-center analysis, we aimed to (1) assess patient-reported QoL and associated factors in working age persons with acute ischemic stroke and (2) identify potential predictors of QoL at 3 months post-stroke.

## Methods

### Participants

In this prospective, observational, cohort study, we invited all persons aged 18–55 years with an acute imaging-proven stroke (either ischemic or hemorrhagic) or cerebral sinus venous thrombosis from the Department of Neurology of the University Clinic Graz to participate in the Graz Stroke in the Young Study (February 2016-August 2020; *N* = 417) (14). The study protocol is shown in the Supplementary Materials.

The study was approved by the ethics committee of the Medical University of Graz. All participants gave written informed consent.

### Health-Related Quality of Life Assessment

We assessed health-related QoL at the acute stage (during initial hospital stay in a clinically stable phase) and at 3 months post-stroke (follow-up, FU) using the EuroQol 5 Dimension - 3 level questionnaire (EQ-5D-3L) (15). The EQ-5D comprises five dimensions (“Mobility,” “Selfcare,” “Usual Activities,” “Pain/Discomfort,” and “Anxiety/Depression”) and three levels (“no problems,” “some problems,” and “severe problems”). We calculated validated index values (*QoL Utility Index*) from these severity levels, with higher values indicating better QoL (1 being best health state). Given that a representative validated Austrian QoL Utility Index is not available, we used the European Utility Index mean value derived from six countries (Germany, Netherlands, France, Spain, UK, and Denmark) (16). Furthermore, the visual analog scale (*EQ VAS*) recording persons' *self-rated health* on a scale ranging from 100 (“best imagined health”) to 0 (“worst imagined health”) was applied (15).

### Clinical and Neuropsychological Assessment

All participants underwent routine neurological examinations and neuropsychological assessments during the initial hospital stay and at a pre-specified FU at 3 months post-stroke.

All but 11 participants undergoing cerebral CT underwent brain MRI. Neurological symptoms, vascular risk factors, stroke severity [National Institutes of Health Stroke Scale (NIHSS), modified Rankin Scale (mRS)], stroke etiology (TOAST criteria) (17), and the affected cerebrovascular territories were assessed in every person. The mRS was evaluated by a trained mRS assessor. Cognitive function was screened using the Montreal Cognitive Assessment (MoCA), while Depression and anxiety were assessed with the Hospital Anxiety and Depression Scale (HADS).

For employed persons with stroke, return to work at FU, duration of sick leave within 3 months post-stroke (days), and self-reported impairment of employment (scale 0 “no impairment”−10 “severe impairment” at work due to stroke) were assessed.

### Statistical Analysis

Demographic, clinical, neuropsychological, and QoL data was analyzed with the Statistical Package of Social Science (SPSS, Version 26). The Shapiro Wilk test was used to assess the normality of data distribution. We performed complete case analysis. We applied Wilcoxon test (for non-normally distributed variables) or paired *t*-tests. We used Spearman correlations to identify associations between QoL and demographics, and clinical and neuropsychological data. We performed two stepwise hierarchical regression models, including demographics (step 1), clinical (NIHSS and mRS at discharge; step 2), and neuropsychological measures (step 3) to identify potential predictors of QoL at FU (EQ self-rated health and Utility Index; Bonferroni-adjusted level of significance: 0.025).

## Results

### Patients

**Figure 1:**
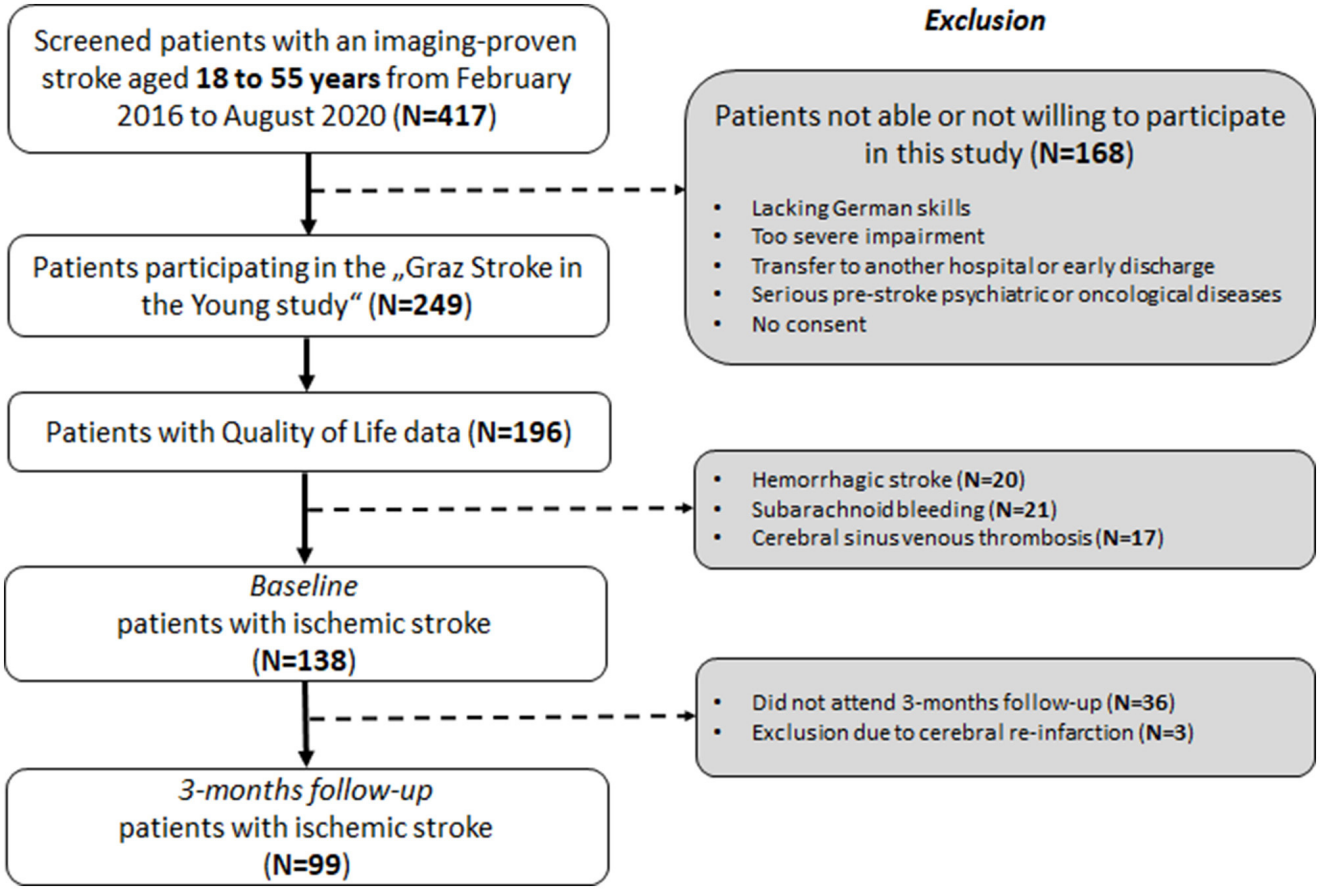
Flowchart of patient selection.

From February 2016 to August 2020, 168 persons with stroke were not able or did not want to participate. From the 249 participating persons, self-reported QoL assessment was available for 196 persons. Given the small sample size of other stroke subtypes and comparability with previous studies (11, 12), we focused on persons suffering from ischemic stroke [*n* = 138 (70%)]. Recruitment details, including a flowchart, are shown in Figure 1.

Regarding baseline (BL) characteristics (Supplementary Table 1), persons participating at FU (*N* = 99) did not differ from those who did not (*N* = 39).

Demographics and clinical and neuroimaging characteristics of the investigated ischemic stroke cohort are presented in Table 1. At baseline, data of 138 persons with stroke (mean age = 43.6 ± 10 years) was available (Figure 1).

**Table 1:** Demographics and clinical and MRI characteristics for the cohort at baseline (BL) (*N* = 138) and for those with available follow-up (FU) at 3 months (*N* = 99) after ischemic stroke.

	***N*** **= 138 baseline**	***N*** **= 99** **3 months FU**
Age (years, SD)	43.6 (10.0)	43.3 (9.9)
Female sex *N* (%)	57 (41%)	40 (40%)
Median NIHSS, admission (IQR; range)	2 (4; 0–32)	2 (4; 0–32)
Median NIHSS, discharge (IQR; range)	0 (1; 0–9)	0 (1; 0–5)
Median mRS; discharge (IQR; range)	1 (2; 0–4)	1 (2; 0–4)
Median duration of hospital stay, days, (IQR; range)	10 (10;4-63)	11 (11;4–63)
Median days to QoL assessment, (IQR; range)	6 (4; 1–16)	6 (4; 1–16)
**Clinical symptoms**, ***N*** **(%)**		
Hemiparesis	63 (46)	47 (47)
Facial weakness	39 (29)	26 (26)
Hemisensory symptoms	52 (38)	36 (36)
Dysarthria	30 (22)	22 (22)
Aphasia	24 (18)	15 (15)
Hemianopia	12 (9)	5 (5)
Headache	37 (27)	8 (8)
Dizziness/vertigo	25 (18)	6 (6)
**Vascular risk factors**, ***N*** **(%)**		
Smoking	64 (47)	45 (45)
Hyperlipidemia	55 (40)	40 (40)
Hypertension	52 (38)	39 (39)
Diabetes	11 (8)	9 (9)
Prior stroke	10 (7)	7 (7)
Prior depression	10 (7)	8 (8)
**Stroke etiology (TOAST)**, ***N*** **(%)**		
Large-artery atherosclerosis	11 (8)	7 (7)
Cardioembolism	34 (25)	23 (23)
Small-vessel occlusion	22 (16)	17 (17)
Other determined etiology	22 (16)	13 (13)
Undetermined etiology	49 (36)	39 (39)
**Affected cerebrovascular territory**, ***N*** (%)*		
Anterior cerebral artery	4 (3)	4 (4)
Middle cerebral artery	74 (54)	51 (51)
Posterior cerebral artery	32 (23)	19 (19)
Vertebrobasilar	41 (30)	36 (36)

* *multiple territories could be affected*.

Sixteen persons (12%) received intravenous thrombolysis, 12 (9%) underwent mechanical thrombectomy, and 9 (7%) received both recanalization therapies.

QoL assessment was mostly performed on day 6 during hospital stay (Table 1). Neuropsychological scores at BL and FU are presented in Supplementary Table 2.

### QOL at Baseline

**Figure 2:**
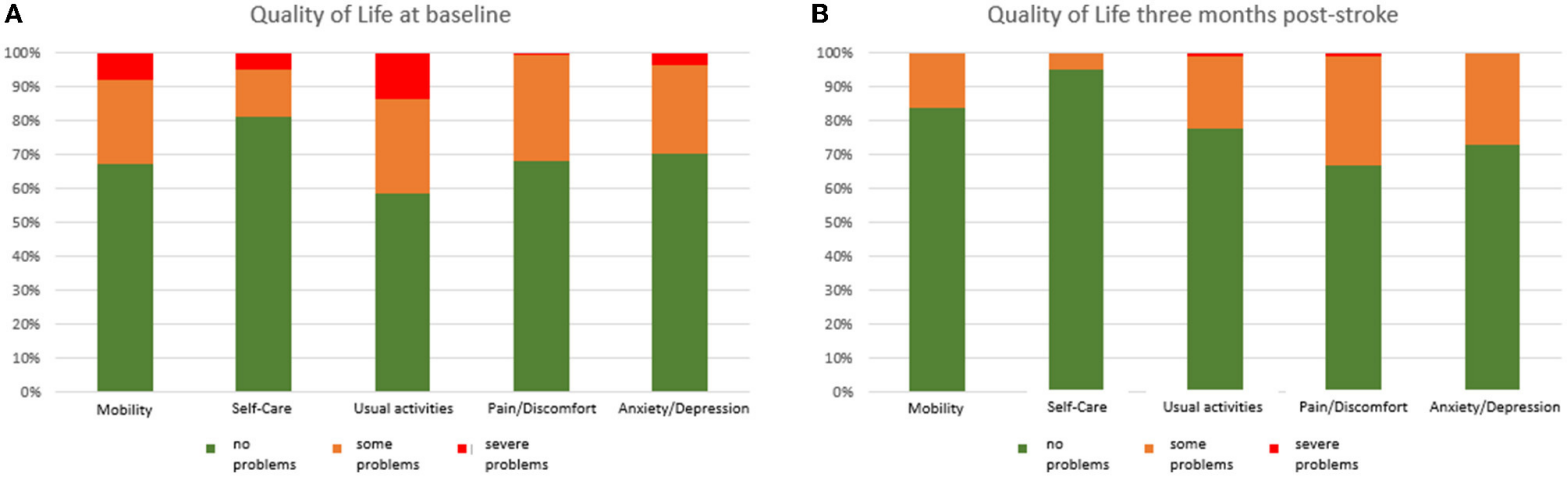
Proportion of problems for each domain at baseline [BL,**(A)**] and follow-up [FU,**(B)**].

At BL, 41.3% of persons with stroke reported serious problems in the domain “Usual Activities,” followed by 32.6% with problems in “Mobility,” and 31.9% reporting “Pain and/or Discomfort” (Figure 2A), while 39.9% reported no problem in any domain.

Median self-rated health (EQ VAS) was 72.5 (IQR = 36.25; range = 0–100) and median QoL Index 0.89 (IQR = 0.34; range = 0.29–1).

Women reported a lower QoL Index compared to men (0.84 ± 0.34 vs.0.91 ± 0.28; *p* = 0.046). Age did not correlate with BL self-rated health or QoL Index. Persons with stroke of undetermined etiology (36%) did not differ from those with known stroke etiology in self-rated health (*p* = 0.433) or QoL Index (*p* = 0.715). Persons with diabetes indicated lower QoL Index (*p* = 0.004; Bonferroni-adjusted level of significance: 0.0083). No further clinical symptoms or vascular risk factors were associated with any QoL measure.

Self-rated health and QoL Index correlated strongly with clinical scores (*r* > 0.5) and moderately with HADS at BL and FU (*r* > 0.3) (Table 2).

**Table 2:** Correlations between health-state and health index and clinical, cognitive, and emotional scores at BL and at 3 months FU.

**BL**	**NIHSS admission**	**NIHSS discharge**	**mRS discharge**	**MOCA**	**HADS Anxiety**	**HADS Depression**
Health state	**-0.2280.007**	**-0.511** **<0.0001**	**-0.532** **<0.0001**	0.1630.056	**-0.387** **<0.0001**	**-0.469** **<0.0001**
Health index	−0.1800.036	**-0.398** **<0.0001**	**-0.456** **<0.0001**	0.1230.151	**-0.417** **<0.0001**	**-0.466** **<0.0001**
**FU**	**Barthel**	**NIHSS**	**mRS**	**MOCA**	**Anxiety**	**Depression**
Health state	**0.360** **<0.0001**	**-0.517** **<0.0001**	**-0.603** **<0.0001**	**0.2680.007**	**-0.467** **<0.0001**	**-0.624** **<0.0001**
Health index	**0.478** **<0.0001**	**-0.534** **<0.0001**	**-0.657** **<0.0001**	0.0700.491	**-0.428** **<0.0001**	**-0.478** **<0.0001**

*MOCA, Montreal Cognitive Assessment; mRS, modified Rankin Scale; NIHSS, National Institute of Health Stroke Scale. Bold values describe significant correlations*.

### QOL at 3 Months Post-stroke

The proportion of persons with serious problems at 3 months post-stroke was 33.3% for the Dimension “Pain/Discomfort,” 27.3% for “Anxiety/Depression,” followed by 22.2% for “Usual Activities” (Figure 2B), while 48.5% reported no problem in any domain.

Three months post-stroke, median self-rated health (EQ VAS) was 80 (IQR = 20; range, 30–100) and Median QoL Index 0.93 (IQR = 0.20; range = 0.41–1). Both measures were strongly correlated (rs = 0.633; *p* < 0.001). Notably, within the group of 79 persons (80%) with mRS scores of 0 (no symptoms) or 1 (no significant disability), 20 persons (25.3%) indicated problems in “Usual Activities,” 18 persons (22.8%) reported some problems in “Anxiety/Depression,” 9 persons (11.4%) reported “Pain/Discomfort,” 5 persons (6.3%) reported some problems in “Mobility,” and one person (1.3%) indicated some problems in “Self-Care.”

Quality of life 3 months post-stroke (self-rated health and QoL Index) did not differ with regard to sex or age, but correlated strongly with clinical scores, followed by strong and moderate (*r* > 0.3) correlations with HADS, and small to moderate (*r* > 0.1) correlations with MOCA scores at FU (Table 2).

Persons with prior depression indicated lower QoL Index values at FU (*p* = 0.001; Bonferroni-adjusted level of significance: 0.0083). No further clinical symptoms or vascular risk factors were significantly associated with any QoL measure.

Out of all initially working persons (*N* = 89; 90%), those who did not return to work (*N* = 48) within 3 months post-stroke reported lower QoL [self-rated health: 75 ± 30 vs. 90 ± 14.5; *p* = 0.002; QoL Index: 0.89 (0.25) vs. 1 (0.07), *p* < 0.0001]. Duration of sick leave correlated with self-rated health (rs = −0.375, *p* < 0.0001) and QoL Index (rs = −0.401, *p* < 0.0001) at FU. Self-reported impairment at work was strongly associated with self-rated health (rs = −0.587, *p* < 0.0001) and QoL Index (rs = −0.553, *p* < 0.0001).

### Predictors of QOL 3 Months Post-stroke

Regarding correlations between baseline scores and QoL at FU, BL mRS and NIHSS correlated with self-rated health (rs = −0.442, *p* < 0.001/rs = −0.448, *p* < 0.001) and Qol Index at FU (rs = −0.573, *p* < 0.001/rs = −0.492, *p* = 0.034).

Baseline MOCA scores correlated with self-rated health (rs = 0.374, *p* < 0.001) and QoL Index at FU (rs = 0.214, *p* = 0.034). Baseline anxiety scores correlated with self-rated health (rs = −0.307, *p* = 0.002) and Qol Index at FU (rs = −0.320, *p* = 0.001). Baseline depression scores correlated with self-rated health (rs = −0.358; *p* < 0.001) and Qol Index at FU (rs = −0.357; *p* < 0.001).

In multivariable analysis, patient-reported self-rated health 3 months post-stroke was predicted by NIHSS at discharge (*R*^2^ = 20.7%; standardized beta = −0.377; *p* < 0.0001) and baseline MOCA score (incremental *R*^2^ = 11.2; standardized beta = 0.357, *p* = 0.001). Together, these showed a variance of 31.9%.

Patient reported QoL Index at FU was predicted by sex (worse for women, *R*^2^ = 5.6%; standardized beta = −.262, *p* = 0.007), NIHSS at discharge (incremental *R*^2^ = 25.5%; standardized beta = −.406; *p* < .0001) and HADS Depression score (incremental *R*^2^ = 4.5%; standardized beta = −.251; *p* = 0.017). Together these showed a variance of 35.6%.

## Discussion

Our findings underline the importance of using patient-reported outcome measures to better capture the diverse sequelae post-stroke. Despite strong associations between routine clinical stroke outcome scores, such as the NIHSS and mRS, and QoL, approximately one-fourth of persons with no significant disability (mRS 0 or 1) indicated some problems in the dimensions “Pain/Discomfort,” and about one-fifth indicated problems in the dimension “Anxiety/Depression” at 3 months post-stroke.

Stroke-induced pain includes a heterogeneous group of conditions, such as pain due to spasticity, headache, and central post-stroke pain (CPSP) (18). In 5.9% of 824 working-age stroke survivors of the Helsinki Young Stroke Registry, persistent CPSP was observed and linked to lower QoL (18). Interestingly, we noticed that persons exclusively reported pain from headache at 3 months post-stroke (Table 1), but, notably, no one had CPSP. This could be explained by our less severely affected stroke population. Post-stroke headache associated with ischemic stroke is poorly understood, only occurring in ~14% of patients (19, 20). This underscores the importance to further explore headache associated with ischemic stroke to inform patient management.

Six persons indicating problems in the domain “Pain/Discomfort” reported to suffer from dizziness or vertigo at FU. Both are common post-stroke, and its multifactorial cause (e.g., visual impairment, sensory impairment, muscle weakness, illusion of movement) makes diagnosis and management challenging (21, 22).

Besides problems in “Pain/Discomfort,” relevant problems in “Anxiety/Depression” were reported 3 months post-stroke. It is noteworthy that according to the HADS, 18% of patients reported mild anxiety and 10% reported mild depression at FU, with only one person reporting to have severe anxiety and depression (scores > 15). Nevertheless, like in our study, working age stroke survivors frequently report feeling anxious, worried, less happy, and slowed down, affecting QoL (6). Due to the independent detrimental effect on stroke outcome, screening for mild depression and anxiety is thus increasingly recommended (6, 11, 23, 24).

Especially for persons clinically classified with an mRS of 0 or 1, assessing self-reported problems in mental health, cognition, and participation seem crucial to truly capture the multidimensional burden post-stroke (6).

In line with a study investigating older persons with stroke (13), both cognition and emotional factors independently contributed to the prediction of QoL and self-rated health at FU from NIHSS. Problems regarding cognition and emotion are highly prevalent in stroke, but receive little attention in clinical practice (25). In older stroke populations, cognitive impairment post-stroke has been shown to be associated with a range of worse outcomes (e.g., worse QoL, less independence, increased likelihood of depression) (26).

Screening all persons with stroke for possible cognitive impairment (e.g., Mini-Mental State Examination or MOCA) or increased emotional burden (e.g., HADS, or patient-reported outcomes, such as the EQ-5D) should be part of the standardized stroke assessment. Early identification and treatment of depression and cognitive impairment may potentially improve QoL among persons with stroke (12).

We found that the female sex was predictive of lower QoL Index scores at FU. A recent study investigating underlying reasons for poorer QoL in women post-stroke identified women's advanced age, more severe strokes, pre-stroke dependency, and post-stroke depression accounting for these sex differences (27). Reasons for lower QoL in working age female patients might differ, considering that women did not differ regarding age (*p* = 0.766), stroke severity (NIHSS; *p* = 0.690), or post-stroke depression (*p* = 0.892) in our sample. This needs to be explored further incorporating socioeconomic aspects in more detail.

Several post-stroke consequences are complexly inter-related. More severe functional impairments might result in higher dependency regarding activities of daily living, potentially leading to more severe depression or less participation. Conversely, more severe depression might lead to less participation, decreased performance in activities of daily living, or rehabilitation engagement. Many of these factors have been shown to influence the likelihood of returning to work, either in combination or independently (28).

In our cohort, persons not returning to work within 3 months post-stroke reported lower QoL. For those working, self-reported impairment at work was strongly associated with lower QoL. Interdisciplinary rehabilitation, including physiotherapeutic *and* psychological treatment, increases the ability to return to work post-stroke and, consequently, improves QoL (28–30).

The inclusion of patient-reported outcomes would not only help to more comprehensively capture stroke outcome (4), but is also recently shown to provide efficiency gains in stroke trials given the use of a “utility weighted mRS” (combining mRS and EQ-5D), leading to reduced sample sizes to detect treatment effects (31).

Several limitations have to be considered when interpreting the results of our study. Firstly, we did not assess fatigue, which was previously found to be associated with QoL post-stroke in working age patients (11, 12). Fatigue and depression are common consequences of stroke and are often inter-related. Both stroke-related symptoms interfere with the rehabilitation process and reduces QoL (32, 33). Secondly, the 3-month FU period might have been too short to adequately assess more long-lasting post-stroke consequences in daily living. Thirdly, we did not assess QoL in age and sex-matched healthy controls over 3 months. However, in addition to self-rated health, we calculated the European Utility Index mean value derived from six countries, which is a well-validated score to assess health-related QoL in clinical populations.

In conclusion, our data suggests that routine assessment of patient-reported outcomes may usefully aid to better capture comprehensive information on post-stroke consequences. The EQ 5D assesses subjective post-stroke problems in <5 min. In addition, such additional screening for cognitive and emotional problems seems promising to optimize individual treatment plans.

## Data Availability Statement

Data that support the findings of this study are available from the corresponding author upon reasonable request.

## Ethics Statement

The studies involving human participants were reviewed and approved by Ethics Committee of the Medical University of Graz. The patients/participants provided their written informed consent to participate in this study.

## Author Contributions

DP, SF-H, CE, and TG contributed to the study conception and design. DP, VF, LB, GB, SH, SE, MK, and CE performed material preparation and data collection. DP performed data analysis. The first draft of the manuscript was written by DP and all authors commented on previous versions of the manuscript. All authors read and approved the final manuscript.

## Conflict of Interest

The authors declare that the research was conducted in the absence of any commercial or financial relationships that could be construed as a potential conflict of interest.

## Publisher's Note

All claims expressed in this article are solely those of the authors and do not necessarily represent those of their affiliated organizations, or those of the publisher, the editors and the reviewers. Any product that may be evaluated in this article, or claim that may be made by its manufacturer, is not guaranteed or endorsed by the publisher.
