# Supplementary material for: Relevance of Cognition and Emotion for Patient-Reported Quality of Life After Stroke in Working Age: An Observational Cohort Study
Source: Front Neurol. 2022 Apr 25;13:869550. doi: 10.3389/fneur.2022.869550 (PMC9081872; doi:10.3389/fneur.2022.869550)
Supplement: Supplementary file 1 [file Data_Sheet_1.docx]

**Supplementary Material**

**Relevance of cognition and emotion for patient-reported quality of life after stroke in working age: an observational cohort study**

**Protocol for the Graz Stroke in the Young Study:**

In this observational study, we invite all patients aged 18–55 years with an acute imaging-proven stroke (either ischemic or hemorrhagic) or cerebral sinus venous thrombosis at the Department of Neurology (University Clinic Graz) to participate during the initial hospital stay and at a pre-specified follow-up at three months post-stroke.

The main research questions of this study are:

1. Assessment of risk factors associated with Stroke in the Young
2. Assessment of functional, motor and neuropsychological impairment (cognition, mood and quality of life) at the acute stage and three months post-stroke
3. Prediction of **stroke outcome** three months post-stroke by demographics, clinical and neuropsychological measures

**BASELINE ASSESSMENT:**

**Clinical assessment:**

NIHSS at baseline, stroke subtype (ischemic, intracerebral bleeding, subarachnoid hemorrhage, cerebral venous thrombosis); if ischemic stroke (TOAST etiology), NIHSS & mRS at hospital discharge, days spent at the hospital. Complications during hospital stay.

**MRI**: stroke subtype, lesion location, preexisting cerebrovascular lesions; features of cerebral small vessel disease including grading of white matter hyperintensities according to the Fazekas score, grade of sulcal and ventricular atrophy, number and location of lacunes and microbleeds.

**Assessment of vascular risk factors/diseases:**

Hypertension, hyperlipidemia, diabetes mellitus, atrial fibrillation, migraine, sleep apnea, coronary heart disease, peripheral vascular disease, venous thrombotic diseases, PFO, smoking, alcohol abuse, depression, epilepsy, cancer.

**Medication**

**Neuropsychological assessment:**

**Cognition**: Screening for global cognition (Montreal Cognitive Assessment), processing speed (Symbol Digit Modalities Test), attention, set-shifting (Comprehensive Trail Making Test 2 & 5), lexical and semantic word fluency (Regensburger Word Fluency Test)

**Mood**: Anxiety and Depression (Hospital anxiety and depression scale)

**QoL**: Quality of life (EuroQol 5 Dimesions)

**FOLLOW-UP ASSESSMENT**

**Clinical assessment:**

NIHSS, mRS, at FU, newly emerged neurological symptoms (yes/no), recurrent imaging-proven stroke (yes/no), Medication,

**Neuropsychological assessment:**

**Cognition**: Screening for global cognition (Montreal Cognitive Assessment), processing speed (Symbol Digit Modalities Test), attention, set-shifting (Comprehensive Trail Making Test 2 & 5), lexical and semantic word fluency (Regensburger Word Fluency Test)

**Mood**: Anxiety and Depression (Hospital anxiety and depression scale)

**QoL**: Quality of life (EuroQol 5 Dimensions)

**Return to Work** after within months (yes/no), amount of hours working post-stroke

**Table S1:** Demographics, clinical and MRI characteristics for the cohort not attending the three months follow-up (N=39) compared to those with available FU at three months (N=99) after ischemic stroke.

|  | **N=39**  **Without FU** | **N=99**  **3 months FU** | **p** |
| --- | --- | --- | --- |
| Age (years, SD) | 44.2 (10.1) | 43.3 (9.9) | 0.627 |
| Female sex N (%) | 17 (44%) | 40 (40%) | 0.732 |
| Median NIHSS, admission (IQR; range) | 2 (3; 0-22) | 2 (4; 0-32) | 0.813 |
| Median NIHSS, discharge (IQR; range) | 1 (1; 0-9) | 0 (1; 0-5) | 0.109 |
| Median mRS; discharge (IQR; range) | 1 (2; 0-4) | 1 (2; 0-4) | 0.808 |
| Median duration of hospital stay, days, (IQR; range) | 10 (6; 4-42) | 11 (11;4-63) | 0.226 |
| Median days to QoL assessment, (IQR; range) | 6 (4; 2-15) | 6 (4; 1-16) | 0.815 |
| **Clinical symptoms, N(%)** |  |  |  |
| Hemiparesis | 16 (41) | 47 (47) | 0.493 |
| Facial weakness | 13 (33) | 26 (26) | 0.535 |
| Hemisensory symptoms | 16 (41) | 36 (36) | 0.611 |
| Dysarthria | 8 (21) | 22 (22) | 0.826 |
| Aphasia | 9 (23) | 15 (15) | 0.269 |
| Hemianopia | 2 (5) | 5 (5) | 0.826 |
| Headache | 4 (10) | 8 (8) | 0.817 |
| Dizziness/vertigo | 3 (8) | 6 (6) | 0.875 |
| **Vascular Risk Factors, N(%)** |  |  |  |
| Smoking | 19 (49) | 45 (45) | 0.729 |
| Hyperlipidemia | 15 (38) | 40 (40) | 0.834 |
| Hypertension | 13 (33) | 39 (39) | 0.508 |
| Diabetes | 2 (5) | 9 (9) | 0.439 |
| Prior Stroke | 3 (8) | 7 (7) | 0.899 |
| Prior depression | 2 (5) | 8 (8) | 0.547 |
| **Stroke etiology (TOAST), N(%)** |  |  |  |
| Large-artery atherosclerosis | 2 (5) | 7 (7) | 0.677 |
| Cardioembolism | 8 (21) | 23 (23) | 0.571 |
| Small-vessel occlusion | 7 (18) | 17 (17) | 0.808 |
| Other determined etiology | 6 (15) | 13 (13) | 0.683 |
| Undetermined etiology | 16 (41) | 39 (39) | 0.891 |
| **Affected cerebrovascular territory, N(%)*** |  |  |  |
| Anterior cerebral artery | 1 (3) | 4 (4) | 0.895 |
| Middle cerebral artery | 21 (54) | 51 (51) | 0.843 |
| Posterior cerebral artery | 9 (23) | 19 (19) | 0.754 |
| Vertebrobasilar | 12 (31) | 36 (36) | 0.698 |

*multiple territories could be affected

**Results**

**Patients**

**Table S2.** Cognition, depression and anxiety scores at baseline (BL) and the three months follow-up (FU). Median scores, interquartile range (IQR) and range are presented.

|  | **BL** | **FU** |
| --- | --- | --- |
| Cognition (MOCA) | 27 (5; 11-30) | 28 (4; 17-30) |
| Depression | 3 (4; 0-15) | 2 (4; 0-16) |
| % with scores >7 indicating at least mild depression | 15.4% | 10.2% |
| Anxiety | 5 (6; 0-16) | 5 (5; 0-16) |
| % with scores >7 indicating at least mild anxiety | 36.8% | 18.4% |

MOCA = Montreal Cognitive Assessment
